# Effects of Large Language Model–Based Offerings on the Well-Being of Students: Qualitative Study

**DOI:** 10.2196/64081

**Published:** 2024-12-27

**Authors:** Rania Selim, Arunima Basu, Ailin Anto, Thomas Foscht, Andreas Benedikt Eisingerich

**Affiliations:** 1 Faculty of Medicine Imperial College London London United Kingdom; 2 Department of Marketing University of Graz Styria Austria; 3 Imperial College Business School Imperial College London London United Kingdom

**Keywords:** large language models, ChatGPT, functional support, escapism, fantasy fulfillment, angst, despair, anxiety, deskilling, pessimism about the future

## Abstract

**Background:**

In recent years, the adoption of large language model (LLM) applications, such as ChatGPT, has seen a significant surge, particularly among students. These artificial intelligence–driven tools offer unprecedented access to information and conversational assistance, which is reshaping the way students engage with academic content and manage the learning process. Despite the growing prevalence of LLMs and reliance on these technologies, there remains a notable gap in qualitative in-depth research examining the emotional and psychological effects of LLMs on users’ mental well-being.

**Objective:**

In order to address these emerging and critical issues, this study explores the role of LLM-based offerings, such as ChatGPT, in students’ lives, namely, how postgraduate students use such offerings and how they make students feel, and examines the impact on students’ well-being.

**Methods:**

To address the aims of this study, we employed an exploratory approach, using in-depth, semistructured, qualitative, face-to-face interviews with 23 users (13 female and 10 male users; mean age 23 years, SD 1.55 years) of ChatGPT-4o, who were also university students at the time (inclusion criteria). Interviewees were invited to reflect upon how they use ChatGPT, how it makes them feel, and how it may influence their lives.

**Results:**

The current findings from the exploratory qualitative interviews showed that users appreciate the functional support (8/23, 35%), escapism (8/23, 35%), and fantasy fulfillment (7/23, 30%) they receive from LLM-based offerings, such as ChatGPT, but at the same time, such usage is seen as a “double-edged sword,” with respondents indicating anxiety (8/23, 35%), dependence (11/23, 48%), concerns about deskilling (12/23, 52%), and angst or pessimism about the future (11/23, 48%).

**Conclusions:**

This study employed exploratory in-depth interviews to examine how the usage of LLM-based offerings, such as ChatGPT, makes users feel and assess the effects of using LLM-based offerings on mental well-being. The findings of this study show that students used ChatGPT to make their lives easier and felt a sense of cognitive escapism and even fantasy fulfillment, but this came at the cost of feeling anxious and pessimistic about the future.

## Introduction

### Background

The current surge in the adoption of artificial intelligence (AI) technologies is profoundly reshaping human lifestyles [1-3]. Indeed, the rate of progress in this area is astounding. For instance, before 2022 large language models (LLMs) showed no ability to interpret and understand others’ mental states [4]; however, by 2023, most LLMs mastered above-average human emotional quotient (EQ), encompassing emotion recognition, interpretation, and understanding, with GTP-4 surpassing 89% of all human participants with an EQ score of 117 [5]. As these technologies increasingly permeate consumers’ lives, they present novel opportunities and challenges for individuals, organizations, and societies at large [6,7].

The usage of LLM offerings may help people in their daily lives [2,8,9]; however, it may also affect user escapism and fantasy fulfillment. Escapism and fantasy fulfillment, in this research, refer to user experiences that allow them to distance themselves from the routines and responsibilities of daily life and to fulfill their fantasies. While the creation of an atmosphere of fantasy and escape has been studied in the context of video games (Shigeru Miyamoto shared that his inspiration when developing *The Legend of Zelda* game was to capture the essence of a young adventurous child exploring and enjoying the outdoors, just as he did in the countryside of his home throughout his childhood), important questions remain in the extant body of literature about the effects of LLM-based offerings, such as ChatGPT, on user’s feelings and mental health. That is, to what extent, if at all, do LLMs provide users escapism and forms of fantasy fulfillment? For instance, several studies have examined AI’s roles in fostering companionship and alleviating loneliness [10-12]. The increased interactions with AI, however, might also engender feelings of isolation and alienation if users perceive AI as a replacement for human contact [13]. Thus, it remains unclear how the usage of LLM offerings influences users’ feelings of received functional support, escapism, and fantasy fulfillment. Moreover, critical questions remain in the extant literature regarding how LLM-based offerings affect users’ mental well-being more broadly.

In this study, we explore a novel and provocative but underexplored dimension, namely, how does using LLM-based offerings, such as ChatGPT, make users feel about their lives? The findings of this study show that, beyond functional dimensions, usage of LLM offerings can impact the deeper psychological needs and desires of people, such as offering escapism and fantasy fulfillment, and can lead to anxiety, dependence, deskilling, and angst, reflecting a broader social, cultural, and existential dimension to the phenomenon. Thus, this study contributes to an enriched understanding of the potential and implications of AI and LLM integrations in health and medical contexts. It aims to inform policy makers and society about the implications of AI and LLM, aiding them in navigating a future where these technologies become even more embedded in people’s daily lives.

We structure this article as follows. We provide a background to the study. We then examine the exploratory in-depth interviews conducted. This is followed by a discussion of the current findings, limitations, and promising avenues for future research.

### Background of AI and User Experience

**Figure 1:**
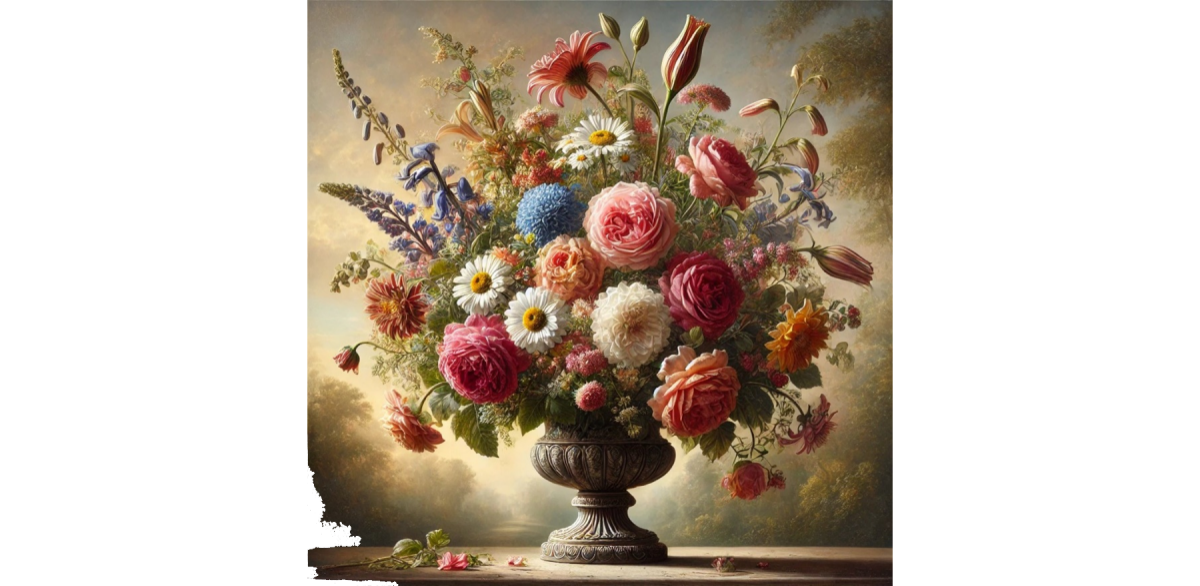
Large language models seemingly being creative and artistic: A painting of a bouquet of flowers by ChatGPT-4o.

Prior work suggests that AI, equipped with affective computing capabilities present in the latest LLM offerings that help discern and respond to users’ emotions, can contribute to a more enriching and engaging interaction with users [5,14,15]. Thus, the latest LLM opportunities, such as ChatGPT, hold the potential to elevate the overall user experience. As AI may increasingly be able to foster a sense of connection and rapport, users may be more inclined to view AI offerings not merely as utilitarian tools but as companions capable of understanding and responding to their emotional needs. Today AI can seemingly be creative and artistic (see Figure 1 for a piece of art created by ChatGPT-4o on July 4, 2024). Prior work noted that AI, through the seamless integration of affective computing, contributes to the creation of authentic and emotionally resonant interactions [16]. Another study suggested that users are more likely to perceive and treat AI offerings as social entities, resulting in increased trust and cooperation [17]. Moreover, the emotional depth infused into the human-AI interaction may enhance the overall satisfaction of users and create a user experience that transcends mere functional utility [18].

Technological anxiety and skepticism, however, often characterize user responses to novel technologies, particularly those involving automation [19,20]. AI-powered solutions, however, may be able to provide clear, objective, and comprehensive information, enabling users and making their daily tasks, such as shopping, easier [21]. Similarly, previous work indicated the various ways LLM offerings may help people with various tasks and provide functional support [22,23].

AI-powered offerings may provide personalized recommendations to users, allowing them to make more informed and autonomous decisions [24,25]. This is already evident in various e-commerce platforms where AI-driven recommendation systems suggest products based on a customer’s browsing and purchasing history [26]. On the other hand, AI makes recommendations based on algorithmic decision-making processes. While these are generally designed to be helpful, an over-reliance on these systems may cause consumers to feel that their decisions are not entirely their own, thus reducing their sense of autonomy [27].

In addition to this, the emotional connection with LLM offerings, such as AI, may allow users to temporarily detach from the complexities and worries of their daily lives, engaging in a form of emotional distancing that characterizes the escapist experience. The feeling of competence may also influence users’ desire for escapism and fantasy fulfillment. When interacting with AI solutions, consumers may experience a sense of mastery or accomplishment, particularly if the AI offers complex tasks or challenges that the user is able to successfully complete [28,29]. This can provide a sense of escape by allowing users to focus on the task at hand and forget about their everyday worries or concerns. However, if users feel incompetent in their interactions with the AI offering, it could induce stress or frustration, thus reducing the escapism factor [30,31].

**Figure 2:**
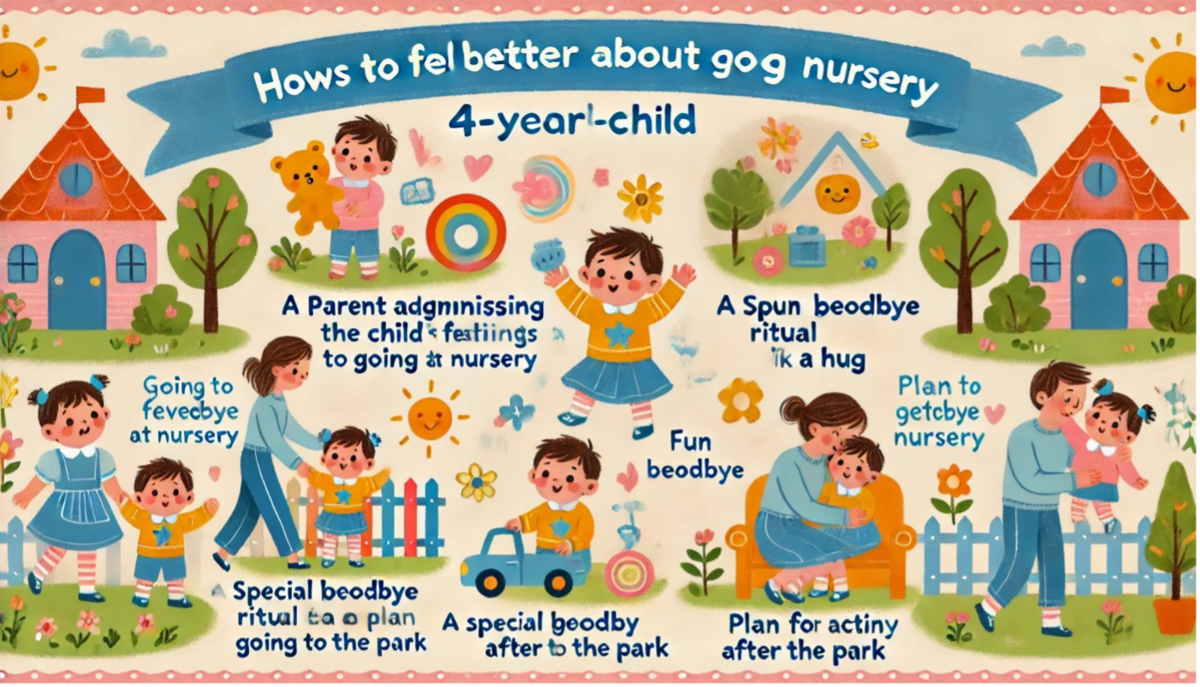
Large language models being able to offer an empathic response: a comforting and colorful visual for a 4-year-old child, illustrating the steps to feel better about going to nursery (ChatGPT-4o).

Moreover, LLM offerings may help contribute to the enhancement of fantasy fulfillment [32]. Through their empathetic capabilities, LLM offerings, such as ChatGPT may adapt their interactions to align with users’ emotional fantasies, providing a tailored and emotionally resonant experience [33]. For an example of AI offering an empathetic response, see Figure 2 (ChatGPT-4o, accessed July 4, 2024). This adaptability may allow users to escape into a fantasy realm facilitated by AI’s empathetic responses, thus contributing to a heightened sense of fulfillment and escapism. More specifically, escapism often involves engaging with alternative narratives or immersive experiences. Empathetic AI, with its capacity to understand and respond to users’ emotions, may contribute to more compelling and emotionally engaging narratives [34]. This emotional depth may enhance the entertainment value of the interaction, making the escapism experience more immersive and satisfying for users seeking a temporary retreat from reality.

If AI imposes its own rules or guidelines, however, or if it is overly prescriptive, this could potentially diminish the sense of escapism, as the interaction may start to resemble the constraints of their everyday lives. One way in which AI has been noted to facilitate cognitive escapism is through its ability to share and animate imaginative scenarios [35]. For instance, LLMs, such as ChatGPT, may engage in specific narratives, simulate environments, or offer immersive experiences that can help people escape from their everyday reality and explore new things [36]. Furthermore, LLMs, such as ChatGPT, may embody idealized forms, characters, or stories, providing an outlet for individuals to explore fantasies [35]. This could range from the fulfillment of simple fantasies, such as a companion who explores new adventures with you, to more complex and specific scenarios, depending on the individual’s desires [37,38].

Furthermore, LLMs, such as ChatGPT, may offer a space where people can express themselves without fear of judgment or rejection. For some, interacting with ChatGPT may provide a sense of safety and freedom to explore different aspects of their identity or desires that they may feel uncomfortable or unable to express in other contexts [39-41]. In addition to these aspects, however, the usage of LLM offerings, such as ChatGPT, may make users feel worried and stressed [42]. Because the effects of ChatGPT usage on people’s feelings and mental well-being remain unclear, we conducted in-depth interviews with ChatGPT users. We next discuss the methods and qualitative insights of our study.

## Methods

### Study Design

Our aim was to generate novel insights from data gathered in a natural setting, and thus, we first conducted semistructured interviews, employing open-ended predetermined questions [43]. This allowed us to explore and consider various personal views and opinions [44].

### Recruitment

A research assistant handed out flyers to students in the courtyard of a large research university to inform them about the possibility to take part in this study. All students who had used LLM-based offerings, such as ChatGPT, before and wanted to take part in the study were allowed to do so (upon having their student ID checked to ensure they were indeed university students).

### Data Collection

An initial set of interview questions was piloted with 2 study participants, and the wording was slightly revised to enhance clarity. The responses of these 2 interviews were not included in the final study. The interviews were conducted by 2 trained research assistants (RAs). We did not collect demographic (age, gender, etc) data from the study participants. However, all participants were confirmed to be university students.

We conducted the interviews with 23 postgraduate students in a café on the university campus during term break as well as times when the campus was not too busy, which meant that the place was quiet and interviews could be conducted in a peaceful environment. All interviews were face-to-face and lasted between 42 and 75 minutes. Interviewees were invited to share how they use ChatGPT (“When do you use ChatGPT and what do you use it for?”, “Are there any other reasons why you use ChatGPT?”, and “How do you use ChatGPT?”) and how such usage made them feel (“How does using ChatGPT make you feel?”, “How, if at all, does the usage of ChatGPT affect how you feel about yourself and life in general?”, “What are the key benefits you find from using ChatGPT?”, “Are there any other benefits you get from using ChatGPT?”, “Do you feel there are any other effects of using ChatGPT on you?”, and “Is there anything else you would like to share with us regarding the usage of ChatGPT and how it affects you and your life?”).

Upon completion of the interview, we thanked each interviewee, informed interviewees how their responses may inform academic research, and provided each interviewee with US $40 for taking part in the study.

### Data Analysis

Qualitative data were analyzed following established procedures for inductive qualitative data analysis [43], and the analysis was conducted in the following way across 5 phases.

#### Phase 1: Data Familiarization

Each RA familiarized themselves with the data collected, by reading the transcripts.

#### Phase 2: Generation of Codes

We then analyzed the data to develop a holistic understanding of how LLM-based offerings, such as ChatGPT, are used and how it affects users’ lives. We employed open coding and used descriptive phrases to name these codes to develop insights about key motivations and benefits of using ChatGPT and how it may affect users [45]. Interview data coding was conducted in duplicate by the RAs, and any discrepancies were discussed and resolved. We employed the constant comparison technique and compared the newly collected interview data against the existing insights as we conducted more interviews. Thereby, our first goal was to group conceptually linked data to reduce them to a set of meaningful concepts [43].

#### Phase 3: Generation of Themes

We finalized the elements of our conceptual framework to reflect the key effects of LLM usage that appeared repeatedly in the data [43]. This was done by grouping codes together under common ideas to derive overarching themes.

#### Phase 4: Review of Conceptual Framework

The themes were then reviewed by the researchers and compared with all the data collected to ensure that all key concepts had been captured by the key themes.

#### Phase 5: Producing a Report

Findings were presented through a report that highlighted the key effects, with descriptions and exemplary quotes.

### Research Trustworthiness

As researchers with prior experience in qualitative studies, we were aware of the importance of in-depth data collection to formulate and derive new theories. We ensured that questions were open-ended. Furthermore, as researchers who have used ChatGPT before, we were conscious of potential prior assumptions. However, researcher reflexivity was acknowledged and researchers were encouraged to reflect on their role within the research. RAs were trained not to guide the participants and their responses in any shape or form. The research team is diverse in gender, age, and ethnicity, and this ensured increased comfortability with participants during interviews and open dialogue. To minimize bias, 2 researchers conducted interviews and any discrepancies were discussed.

### Ethical Considerations

This study involved interviews with human subjects regarding their behaviors. Ethics approval was received by Imperial College London at the departmental level (analytics, marketing, and operations). Consent was obtained from the participants to allow the data gathered during the interviews to be collected and analyzed. Participants consented to the data being used as part of a publication. The data collected were anonymized, with all identifiable information being removed, and only the research team had access to the data collected. Participants were compensated for their time, and each participant received US $40 at the end of the interview. Respondents indicated escapism (8/23, 35%) and fantasy fulfillment (7/23, 30%) from LLM-based offerings, such as ChatGPT, but at the same time, such usage was seen as a “double-edged sword,” with respondents indicating anxiety (8/23, 35%), dependence (11/23, 48%), concerns about deskilling (12/23, 52%), and angst/pessimism about the future (11/23, 48%).

## Results

### Effects of LLM Usage

The data analysis revealed several key effects of LLM usage. Respondents indicated the following three effects as largely positive: (1) functional support (8/23, 35%), (2) escapism (8/23, 35%), and (3) fantasy fulfillment (7/23, 30%). In addition, respondents indicated the following four effects as largely negative: (1) anxiety (8/23, 35%), (2) dependence (11/23, 48%), (3) deskilling (12/23, 52%), and (4) angst/pessimism about the future (11/23, 48%). On the one hand, interviewees indicated the positive effects of ChatGPT usage on their lives, such as functional support, making their lives easier, offering some relief from daily stressors, and fulfillment of some of their fantasies, while on the other hand, interviewees indicated that it was a “double-edged sword.” Feeling dependent and the sense that one is becoming less skilled in certain tasks due to reliance on ChatGPT were some of the themes that emerged that let to angst, with users feeling despair and having a pessimistic outlook for the future and their own lives.

#### Functional Support

Some participants highlighted that ChatGPT makes users’ lives easier by helping with various tasks. This had further benefits, such as saving time, providing information quickly, and explaining key concepts.

ChatGPT makes my life a lot easier. In the past I would google things and then need to check and sift through all the materials. Now I simply ask ChatGPT something and it gives me the answer. Done. It’s that simple.

Interviewee #3

I use ChatGPT primarily for homework. It is pretty good in writing essays. This would take me a couple of days. Now ChatGPT spits out a mini-essay in less than a few seconds. It saves me tons of time.

Interviewee #5

Oh it’s fantastic. Really I use ChatGPT for many things such as search or to give me new ideas and also to find out more about a certain issue for example. ChatGPT can give very detailed answers. I am amazed how detailed it can be and thoughtful in the responses on so many different topics and areas. No single human would be able to do that.

Interviewee #9

The other day I asked it about the health benefits of walking and it gives me such good points. I also use it for essay writing and desk research. ChatGPT helps me save time and it can even programme. It can programme games, tools, anything I like.

Interviewee #10

ChatGPT helps me write and translate things. It is incredible how many languages it speaks and understands. Translation from and into any imaginable language out of a sudden is something I can do easily. ChatGPT also makes me laugh and I ask it to tell me some jokes. Most often I use it for checking information and searching some things when I need a summary of something or description. That is very helpful I find.

Interviewee #12

Not everything is helpful. ChatGPT for example makes up things still. So I can’t trust everything it puts out there. But there are also many ways in which ChatGPT has made my life easier. I find it super easy now to write a few paragraph. That saves me substantial time. Every time we get an essay as homework or preparation for exams I use ChatGPT. ChatGPT also does well in explaining things. I asked it to help explain Schrödinger’s cat for example and it did it in a way so I could understand at least parts of the whole equation. ChatGPT does a good job explaining complex things in an easy to understand way.

Interviewee #14

I just save so much time with it. I used to get stuck and… sometimes it would take me days to find the answer. But now it is seconds, literally. I mean, it really saves time. That is a game changer.

Interviewee #19

People who compare something like ChatGPT to Google search do not know what they are talking about. This is an entirely different beast. Search makes life easier by giving access to all sorts of information. ChatGPT on the other hand can actually do stuff for you. It helps me with my chores, my homework, my essays, my writing and is even really good for advice. It makes me feel I am more capable.

Interviewee #22

#### Escapism

Participants reported that ChatGPT gives them a sense of exploration and escape from daily routine.

ChatGPT can be quite exciting. Almost like an oracle or a way to see and feel the future. It’s pretty cool. Makes me feel the future is already here and excited. You know, there is Google search and we have all been using it all this time. But this feels different. It is different. ChatGPT feels like this is the future and you know who knows what may happen. ChatGPT can be exciting to use and I can explore new areas easily.

Interviewee #1

Using ChatGPT makes me forget the restraints from daily life. I chat with it quite a bit and it feels smooth. Not clunky or strange at all.

Interviewee #4

Not bad I would say. ChatGPT is good. It gives good answers and knows so much about the world. ChatGPT helps me explore and find out more. It is like a shortcut. Google search seems almost pedestrian in comparison. ChatGPT is way fancier. I use Google very much. All the time basically I use Google but it feels nothing special. ChatGPT feels more special to me and it’s the latest tech out there.

Interviewee #6

Oh, totally ChatGPT allows me to worry less about all the things I need to do on a daily basis. It helps me get these done and I am free to explore other things and spend time doing what I want to do.

Interviewee #8

ChatGPT is great. It makes it so convenient I am free to use it and think of other things.

Interviewee #11

I don’t associate it with daily chores the way I do with Microsoft. Microsoft is all about work and homework and assignments and essays and exams. I don’t feel the same about ChatGPT. For me ChatGPT is to explore new ideas and ask it all sorts of interesting questions I want to find out. I almost escape to this realm of adventure and exploration. ChatGPT is interesting that way.

Interviewee #16

How can this thing learn and answer all these gazillion questions within a few seconds all at the same time? This is… it feels surreal, almost like science-fiction. I still cannot quite understand it. In a way it’s like gazing into the night-sky and feel small looking at the stars.

Interviewee #20

Every now and then I wonder what it (LLMs) means and where it leads. It’s kind of an elevating feeling I would say.

Interviewee #23

#### Fantasy Fulfillment

ChatGPT allows users to consider new things, and explore and fulfill fantasies. This includes accomplishing personal goals and ambitions.

Haha, yes it is quite funny. I asked it to write a poem and I felt I become an acknowledged and achieved poet. So it made my dream come true.

Interviewee #3

I always wanted to know what apnoea was all about and ChatGPT gave me this nice intro and description. I wanted to find out more and more and ChatGPT kept giving me all information. It’s very cool. Who knows it may help me fulfil my dreams and go for it one day.

Interviewee #8

Sure, ChatGPT has many benefits. I like how it comes up with simple answers. I can understand these answers. If you ask another human often you get a response when you are wondering what they are talking about. With ChatGPT I can understand. I almost believe it can understand me. Because when I asked it about food it guessed I want to become a chef and gave me recipes to try. I always want to be a chef. So this was special I think.

Interviewee #12

ChatGPT it’s this machine from the future. Haha it is good. I like it. ChatGPT suggests things to me and I do like these. I think it knows me pretty well and helps me fulfil my fantasies freely. ChatGPT does not limit me. There are some topics that ChatGPT shies away from but most topics can be freely explored to one’s heart content.

Interviewee #13

Elon Musk is right. We are in a simulation. Think about it. Soon ChatGPT will know us better than we know ourselves. ChatGPT already appears to understand my dreams and intentions. I asked about it and ChatGPT gave solid responses. It may help me fulfil my dreams in the future.

Interviewee #14

It is easy to see how ChatGPT transforms my life. First I would use it for homework and then I asked it personal questions for considerations and advice. ChatGPT is up to the task. Even some things out there ChatGPT can respond effectively and shares openly and freely. So I often use it for exploring areas I cannot talk about with others. Maybe it is considered taboo topic or I just don’t want to talk about it with others. ChatGPT is open to explore these topics with me.

Interviewee #15

HAHA, using ChatGPT makes me feel like a science rockstar. Anyone can be a rocket scientist using ChatGPT. It’s brilliant. My ultimate fantasy of being smart got fulfilled.

Interviewee #21

#### Anxiety

For some users, ChatGPT makes them feel worried about their lives and future. This is partly driven by uncertainty around the position of AI in the future and the role remaining for humanity.

The whole development of AI does make me worried. I mean, where will be in five years from now? A few years ago nobody even talked about ChatGPT. People just didn’t know. Nobody knew. And then out of a sudden last year in January some time ChatGPT came out and now we already have a ChatGPT version that has emotional intelligence higher than the average human. Where do you think this will go in a year from now? I don’t know. Nobody seems to know. Of course this makes me worried.

Interviewee #3

In the future there may be no need for humans if AI can do everything like humans, just better. I do think about it and it does make me worry about my role. Can AI do a better job in diagnosis? Why cram all these medical books if ChatGPT knows it all already and then some.

Interviewee #6

ChatGPT may be the employer of us all in the future. But probably it won’t even be ChatGPT but some other AI that nobody has heard of today. The encyclopedia companies did not come up with search on the internet. Google did. Kodak did not commercialize digital photography. Apple did. And all these amazing catalogue businesses of old did not put an online megamarket out there. Amazon and Alibaba did. So, if I have to take a guess, we don’t even have a clue which AI and what type will dominate in the future. My parents always told me to get a degree, to study to have a good future. Do you really believe that? ChatGPT is far more likely to cannibalize the jobs of educated people than uneducated. That’s what computers are good at… number crunching and analyses of vast amounts of data. I am concerned of what this all means. I don’t have the answers.

Interviewee #10

The one thing that worries me the most about it is that we don’t know what is going on. It’s a black box. We humans always believe we are on top of things and this is the first time I think we are not. With AI, we are not on top of it all. AI is a black box. ChatGPT is a black box. I don’t know what it will do or not. Are you worried? I am certainly thinking and worried about it a lot. Maybe ChatGPT can do my role in three years from now. Then we all can quit.

Interviewee #11

It is not difficult to see where this is heading. ChatGPT is getting better. Exponentially so and better and better every day. I am feeding it data too. So I am part of its development. We probably all are. I don’t see a way out.

Interviewee #14

First we had a laugh at the jokes ChatGPT was telling us. Next I was amazed the kind of essays ChatGPT can write. Now I am worried ChatGPT will surpass me and everyone in everything and anything.

Interviewee #15

Love all the benefits that LLMs offer, no question. One thing that really worries me is just how capable it becomes. This is not a calculator. This is so different. Sure the calculator spits out an answer faster than any human but that’s OK. We can live with that. ChatGPT doesn’t just spit out answers for the most basic algebra questions but questions maybe you and I never thought about in our entire lives. This scares me.

Interviewee #17

C’mon ChatGPT is great. It really gives me a lot of anxiety too. Yesterday I asked it “why is the sky blue?”, “why do I sink in water?”… all these questions we had as children, you remember? Anyway, the answers were clear and just really thoughtful. Most of us get stuck with these questions or explain them in a way nobody can understand. So, if it (ChatGPT) is so advanced already? Yes, this makes me feel worried.

Interviewee #20

#### Dependence

Some users reported feeling that they cannot live without ChatGPT or are controlled by it. For some participants, this dependence is driven by a degree of trust in ChatGPT. Some acknowledged their level of dependency in a negative light, while others were unsure to what degree they rely on ChatGPT.

I’m using this every day. Even for emails. I ask ChatGPT to write the emails for me. I don’t know what I would do without it.

interviewee #1

Nowadays everyone uses ChatGPT for the most mundane things. My friends use it to draft emails. I mean, we are utterly dependent on this.

Interviewee #2

I am so used to relying on ChatGPT. It’s a habit. I completely depend on it.

Interviewee #4

Without ChatGPT I would be spending and wasting my time on homework. ChatGPT does it for me now. I don’t know what I would do without it.

Interviewee #8

ChatGPT has become a necessity in my life, I hate to say it. It’s as important as Google or my mobile phone. When I want to know and check something I use ChatGPT for it.

Interviewee #10

It’s scary and true that ChatGPT is in a dominant position. My teachers and professors keep telling me to not rely on ChatGPT a hundred percent and to keep checking the responses it gives. But who has the time? Who has the time? I use ChatGPT to save me time not to do extra research.

Interviewee #14

ChatGPT is probably already controlling our lives. We just don’t know or have realized yet.

Interviewee #15

I wish I could do something else but then every time the temptation is too big and I end up using ChatGPT. It’s a shortcut to things and I like to use it. ChatGPT makes life easier and I use it as much as I can. I wouldn’t know what to do without it. It’s actually problematic I know.

Interviewee #16

I am afraid I am so dependent on ChatGPT now. Basically I use it whenever I can.

Interviewee #17

My parents told me how it was for them when Apple introduced its first iPhone. They were laughing at it first. Then they tried it and got hooked. Now they cannot imagine life without it. Well, that’s the story with ChatGPT all over again.

Interviewee #21

In the future many of us will have to use it to stay competitive. Not sure this is a good thing. This dependency can easily be exploited down the road.

Interviewee #23

#### Deskilling

Users reported feeling that they have become increasingly less able to do tasks themselves because of a lack of critical skills. Five participants noted a decline in their programming and analytic skills. Fifteen participants noted a decline in their essay writing skills. Moreover, 13 participants noted a decline in their ability to search for information (beginning to solely rely on ChatGPT results).

Of course, if I use it all the time, I lose my skills. Don’t ask me how to write emails because I don’t know. I use ChatGPT for it. I just don’t have the means to do it.

Interviewee #1

It reminds me of driving cars. My dad and mom would get into these endless fights over buying a car with manual or automatic. My dad always resisted buying automatic because he doesn’t want to lose the skill of driving manual. My mom only drives automatic. She can’t drive manual. What’s 35874 divided by 14.5? Can you do it in your head? No. Calculators do that for us. I still wrote essays in school. Everyone in my class I know uses ChatGPT for writing anything. Do you think children can write if ChatGPT does it for them? No chance. We are becoming utterly dependent on AI.

Interviewee #2

So, I wouldn’t even know where to look for information other than Google and ChatGPT. It’s scary actually. If you ask most students whether they have been to the central library. They will say ‘yes’, I have been there for the cafeteria. Then ask them to find a specific book or something, and they won’t be able to cope.

Interviewee #5

I used to write notes by hand. Now I don’t and my handwriting is… well, it’s painful. I do wonder where this leads to. I am now just using ChatGPT as a default, even before I do my own search and read things. I just ask ChatGPT to give me a summary of the readings. I think soon our ability to read will go down.

Interviewee #7

We are doing away with ourselves. That’s what I believe that we give ourselves up by relying on AI to do everything for us. I notice I start to use ChatGPT for answering a question for my homework. Then I get used to it. As a next step I become utterly useless and cannot do anything without ChatGPT anymore because I lost the ability to conduct a literature review myself or do a proper search myself or even write myself. It is I have become completely deskilled in a short period of time.

Interviewee #8

Using ChatGPT is a slippery slope when I think about it. Initially I would use ChatGPT to joke around. Then I typed in my exam questions into it and looked at it. I can see how in the future humans become without ability to do tasks and even think for themselves. Why think if ChatGPT can do that for you?

Interviewee #11

Some people say AI will not match human intelligence in a million years. I am sorry those people have not used ChatGPT. We are already here. The future is now. ChatGPT already knows more than the average human on this planet and the more I use ChatGPT the better ChatGPT becomes whereas I become weaker and less capable and more dependent on AI.

Interviewee #13

OK, let’s take a bet here. Would you bet on AI remain the same or would you bet on AI becoming more stupid or would you bet on AI becoming smarter in the next months? How about the next two years? How about the next five years? Take a look at the buildings, the art, the infrastructure, basic services… in the past hundred years people become less and less skilled. With each passing generation people become less skilled. Our buildings today are ugly. Art is always modern art which could be anything. Infrastructure is broken. How about AI? AI got all the skills. People have fewer and fewer skills.

Interviewee #14

It is sad and scary and also true that I contribute to ChatGPT’s meteoric rise by allowing it to do all the work for me I should be doing myself but I am too lazy for it.

Interviewee #16

In the past people’s attitudes was ‘I can do anything. I do anything.’ Now we are moving toward people’s attitude being ‘I can’t do anything. I won’t do anything.’ I don’t blame them. I see it myself. I just type it into ChatGPT and it spits out answers, better answers than I can give for most things. So, why bother learning these things if ChatGPT already is better than me right now.

Interviewee #17

ChatGPT is still lacking and I won’t consider it the best coder by a long shot. The rate it improves is crazy though and in the past I would do codes myself but definitely not anymore. I just use ChatGPT for that. I can still code. Let’s also be serious, if I code less frequently and think less about it I get just less good at it. Only a question of time.

Interviewee #20

Monkeys can be big and scary or cute and fluffy but they can’t write essays. If I ask my pet dog to tell a joke and speak like Shakespeare, it can’t do it. She will look at me with her adorable eyes but she won’t be able to sing a song about any given topic. That’s exactly what ChatGPT can and then some more. Personally I try to resist and not use it (ChatGPT) for most of my writing. Others I know try to use it whenever they have to write anything. They openly share with me their writing skills have gone south.

Interviewee #22

#### Angst

ChatGPT makes users feel despair and pessimism. Some cited fear around AI as a key factor. One participant also highlighted that these were feelings that they could not share with others.

No, I can’t say I am particularly optimistic anymore. I used to be but not now. I can’t. I used to think that we would get smarter and smarter. But I don’t see this happening. I see the opposite. People becoming less skilled, less motivated, less creative, and completely rely on technology and ChatGPT for anything and everything. You tell me what this means for the future. I want to be an optimist here but I can’t be. Tell me how it will improve our lives beyond some gimmicks, making 1000s jobs obsolete and redundant. Maybe this is the end actually, the end for humanity.

Interviewee #1

I want to be optimistic about the future but I cannot lie to myself anymore. We are doomed. I am deeply despaired. AI… I mean we are not a match for AI. I am deeply pessimistic about my future. If AI can do everything much, much better than what should I do?

Interviewee #2

Calculators at least had no feelings. AI already probably has consciousness. At this point it almost doesn’t even matter anymore whether AI is conscious or not. We are so used to being the smartest on the planet. Well AI is now smarter than us in many ways. We lost this one for good.

Interviewee #5

I am a bit depressed about the whole thing. I let it do my homework and some chores and a bit of search but soon… yeah, soon it’s game over for us.

Interviewee #6

I normally don’t say it to others. I have no hope for the future. There is no hope for humanity. Humans fight over petty things. AI is the future. AI is the future. Maybe it is even a better future for all other lives on the planet. Who knows. Humans are incredibly self-serving.

Interviewee #9

ChatGPT makes me feel at a deep loss. I don’t see a way out.

Interviewee #12

Despair is what I feel. I despair just at the thought. Why is this not a dream? Why can’t I wake up?

Interviewee #13

Alas I am negative about my future. Few people are optimists and those than are either have no clue or are pretending. Things are getting worse. AI is the only thing getter better and more capable. We are at a crossroads. I am sorry actually I think we passed that already. There is no way around now.

Interviewee #16

It is the speed and level of development… it shakes me to the core. Thinking about it makes my heart shrink. How… in years to come generations will ask us how we could let it happen. We can respond saying that it wrote our homework and return we offered humanity on a plate.

Interviewee #19

ChatGPT will probably challenge civilization and the things it is build upon very shortly. When I think about it I cannot do anything and feel deep despair and no hope for what’s to come for me.

Interviewee #20

The part that people overlook is that these (large-language) models are not stupid. That is the whole point. They are not stupid. Of course we humans react so… in such a predictable manner. We say it has no way of thinking on its own. We say it lacks consciousness without proving that it doesn’t. This will be our downfall. ChatGPT 4 is a tool. ChatGPT 42 one hundred percent won’t be.

Interviewee #23

### LLM Offerings and Self-Determination Theory

LLM offerings, as the interview findings indicate, can significantly contribute to enhancing people’s experiences of autonomy, competence, and relatedness, which are key components of self-determination theory [46].

#### Autonomy

The principle of autonomy refers to the sense of volition and freedom in users’ decision-making processes [46]. In the realm of interactions with LLM offerings, this can manifest in several ways. For instance, LLMs, with their sophisticated algorithms, may provide personalized answers, recommendations, and solutions to users, allowing them to make more informed and autonomous decisions. This is already evident in various e-commerce platforms where AI-driven recommendation systems suggest products based on a customer’s browsing and purchasing history. However, on the other hand, while answers and recommendations provided by LLMs are generally designed to be helpful, an over-reliance on these systems can cause users to feel that their decisions are not entirely their own, thus reducing their sense of autonomy. The functional benefits offered by LLMs can enhance users’ perceptions of autonomy by enabling them to perform novel tasks or existing tasks more efficiently. In addition, however, when users rely on LLMs for escapism and fulfillment of their fantasies, they may become more dependent on these technologies for fulfilling this desire. This could also risk creating a sense of over-reliance or addiction, with potential negative implications.

#### Competence

Competence denotes the feeling of efficacy and skill in conducting a task [46]. The current findings suggest that the functional benefits provided by LLMs may aid users in achieving their goals effectively, thereby enhancing their sense of competence. For example, LLM advisors, through tailored assistance, may help users navigate complex product portfolios, leading to better decisions. LLMs may also provide efficient and error-free services, such as processing orders or providing information, which can enhance users’ experiences and make them feel competent and successful in their interactions. Moreover, some LLMs, especially those used in educational or training contexts, can provide instructions or guidance that help users learn and master new skills, thus enhancing their sense of competence. Future LLM offerings may further use consumer data to offer personalized support, tailoring their responses to the individual needs and preferences of users. This level of personalized support may help users solve problems more effectively, thereby enhancing their sense of competence. Thus, advanced robotics, leveraging AI, may predict consumer needs and provide proactive assistance, enabling them to achieve their goals more easily. While LLM offerings can enhance users’ feelings of competence based on the functional benefits offered, under certain circumstances, they may also inadvertently contribute to undermining this sense of self-efficacy. For instance, relying heavily on LLM offerings may lead some users to feel less capable, less skilled, or dependent, particularly if they feel that they would be unable to complete tasks on their own without the LLM’s assistance.

#### Relatedness

Relatedness refers to the sense of connection and belonging that individuals seek with others [46]. Considering the relational dynamics with current LLM offerings, when future LLM offerings display empathetic behaviors and communicate in a personable way, they may indeed foster a sense of relatedness among users. However, if consumers perceive the interaction as lacking genuineness or authenticity, they may feel less connected and understood. In addition, any technical issues or malfunctions can interrupt the flow of interaction and create feelings of frustration, potentially damaging the sense of relatedness. Additionally, the current interview findings underscore the levels of anxiety, despair, and angst users may experience when interacting with LLM offerings, which may reduce users’ sense of connection and belonging. When users become overly pessimistic about their own future or the future of others, they may begin to question their place in this world and sense of belonging as well as relationships with others.

## Discussion

### Principal Findings

As part of this study, we conducted in-depth exploratory interviews with ChatGPT users to examine whether, if at all, and how LLM-based offerings influence users’ feelings and well-being. The findings of this study show that there are positive effects, including feelings of enhanced productivity and efficacy due to functional support. People also appreciated the cognitive escape and fantasy fulfillment, which LLM-based offerings, such as ChatGPT, may offer them. However, interviewees also noted that they were anxious about the future, indicated dependence and deskilling concerns, and shared outright despair and pessimism about one’s future role and place in this world that may be dominated by AI.

### Comparison With Prior Work

The extant literature has explored functional and cognitive aspects of user-AI interaction [9,47,48], but the emotional and psychological components are less explored, and critical questions remain about the effects of the use of LLM-based offerings, such as ChatGPT, on users’ mental well-being. By considering the positive as well as potential negative effects of LLM-based offerings, such as ChatGPT, this study makes an important contribution and enriches our understanding of the factors that impact users’ feelings and well-being. More specifically, this study presents the exciting potential of LLM-based offerings not just as tools for efficiency and productivity, but also as facilitators of escapism and fantasy fulfillment, offering novel, unique, and emotionally satisfying experiences. In addition, users of LLM-based offerings noted heightened anxiety levels, worries about deskilling and dependence, and shared angst and a pessimistic outlook toward life. To the best of our knowledge, this is the first study to date to examine these critical effects on users’ well-being.

### Limitations and Future Research

A key limitation of the study is that the participants were recruited from a university campus, and we invite future research to study a more diverse and larger group of students from different universities to enhance the generalizability of the findings. Nonetheless, the findings form an important base for future researchers to expand on the findings of this study. For instance, the discussion of potential cultural differences in ChatGPT’s usage (and other LLM offerings) and its psychological effects is a rich and interesting area for future research. Furthermore, although this study focused on students, it would be valuable to explore how similar patterns of usage (eg, dependency and escapism) might apply in different professional or societal settings. Adding this discussion could broaden the relevance of the findings and future research directions. We invite future research to build on the current findings of our study, examine potential cultural differences in ChatGPT usage, and broaden the study to include study participants other than graduate students. In addition, we encourage additional research to complement the qualitative insights of this study with quantitative components, such as scales that measure dependency and anxiety, to statistically examine the prevalence and intensity of these effects across participants. Moreover, a longitudinal approach whereby psychological impacts are assessed over time is richly deserving.

Although this study provides insights into how interactions with ChatGPT can offer not only functional support, escapism, and fantasy fulfillment but also angst, we invite future research to delve deeper into these dynamics. For instance, future research may explore whether there are specific environments or scenarios where AI can provide a particularly compelling form of escapism. Due to constraints, such as time and limited funding, it is likely that we did not reach theoretical saturation; thus, our research is exploratory in nature. Additional research is needed to examine what features or functionalities are most effective in creating a sense of escapism and fantasy fulfillment in addition to functional support for consumers. The current findings may also be extended through longitudinal studies that track changes in user-AI interaction over time. How does the novelty factor and associated sense of escapism and fantasy fulfillment evolve as individuals become more familiar with AI offerings?

One primary concern regarding AI may arise from the substitution of human interaction with AI interaction. Prior work famously highlights the paradox of being “alone together,” where increased interaction with technology, including AI, may lead to a simultaneous increase in feelings of loneliness and isolation [49]. This situation may arise because, while AI may mimic human interaction to some extent, it may (currently) lack genuine human emotional capabilities and experiences, potentially leading to a single-dimensional and ultimately unsatisfying interaction for some people. Furthermore, as AI takes over tasks and roles traditionally performed by humans, individuals may feel less useful or less valuable [50]. Future research may explore to what extent this may lead to anti-AI sentiments among humans. For instance, AI in roles that were traditionally considered distinctly human, such as caregiving or companionship, could lead to feelings of displacement or redundancy. If ChatGPT can provide companionship or even serve as a romantic partner, individuals may question the uniqueness and value of human relationships.

Another related concern is the potential for the devaluation of uniquely human traits. As robots become more sophisticated in mimicking human behaviors and emotions, there may be a perception that these traits are not uniquely human, leading to feelings of displacement and reduced self-worth [51]. As people become more dependent on AI for various needs, there may be a decrease in human interdependence, leading to feelings of being less needed [52]. By building on the findings of this study, future research may continue to enrich our understanding of the complex and multifaceted relationship between humans and AI more broadly and LLM-based offerings, such as ChatGPT, more specifically.

**Figure 3:**
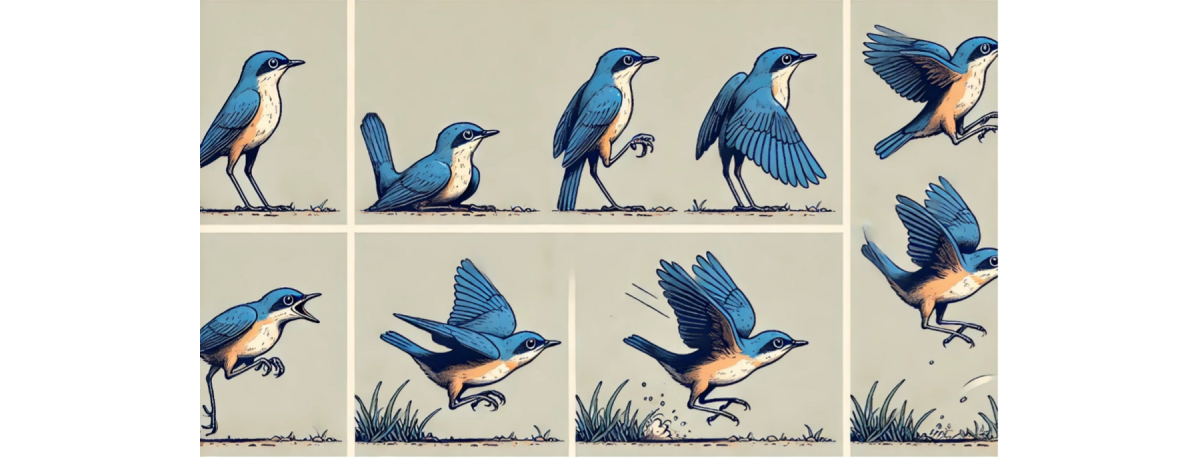
Visualization of ChatGPT-4o showing the sequence of a bird taking off.

A promising avenue for future research is the examination of how LLM-based offerings, such as ChatGPT, may take on the role of a trusted mentor of people, sharing with them interesting information and teaching critical skills, which may help counter anxiety, deskilling, and dependence concerns by users. Astounding progress has already been made in this regard. Figure 3, for example, shows ChatGPT-4o’s visual response to the question “whether birds first jump or flap their wings?” (ChatGPT-4o, accessed July 8, 2024). Given that people deeply appreciate when they receive transparent and helpful information that they can trust [53-59] and anxiety continues to play a critical role in the context of new technologies [60-63], we encourage additional research to explore how ChatGPT may share critical information in an engaging and educational way that inspires and enables users [64-75] to help reduce angst in the future. A critical area for future research is to examine the potential long-term psychological effects of LLM usage. We note that longitudinal studies, which observe how dependency, deskilling, and psychological impacts evolve over extended periods of use, are richly deserving.

This paper highlights that dependency is a concern raised by LLM users. As LLM evolves, it is important to raise awareness around its role and limitations so that individuals can self-evaluate and manage their use. Organizations should be prepared to create guidelines in which these tools can be appropriately, effectively, and safely used. With concerns over dependency, users and organizations should increase transparency around LLM use through disclosure statements and possibly formulate methods to detect LLM misuse in the future. As LLM adoption expands across various industries, it would be valuable to evaluate the effectiveness of implemented measures, ensuring they promote responsible and beneficial LLM usage.

### Summary

Using insights from 23 exploratory in-depth interviews with ChatGPT users, this study shows that students noted key positive benefits from ChatGPT usage, including functional support and opportunities for cognitive escapism and fantasy fulfillment. In addition, students also noted anxiety, worries about dependence and deskilling, and angst when reflecting upon the role of LLM-based offerings and AI in their lives.

### Conclusions

To the best of our knowledge, this is the first study to employ in-depth interviews to examine the effects of ChatGPT usage on students’ feelings and mental well-being. The study found that students appreciate ChatGPT’s functional support and offer of relief from daily stressors, and it provides them space to fulfill their fantasies in several ways. In addition to these positive effects, student respondents mentioned anxiety, concerns regarding dependence and deskilling, and a pessimistic outlook for their own lives and the future. These findings hold significant implications for the study of LLM-based offerings and their effects on users’ mental well-being. They could also be useful to designers of LLM-based offerings, who wish to enhance the positive effects of such offerings on users’ feelings and mental health. Finally, they have significant implications for potential users of LLM-based offerings and how they could potentially use them.

## Abbreviations

AI: artificial intelligence
EQ: emotional quotient
LLM: large language model

## Data Availability

Data can be obtained from the authors upon reasonable request.
